# Combining machine learning and quantum mechanics yields more chemically aware molecular descriptors for medicinal chemistry applications

**DOI:** 10.1002/jcc.26737

**Published:** 2021-08-19

**Authors:** Sara Tortorella, Emanuele Carosati, Giulia Sorbi, Giovanni Bocci, Simon Cross, Gabriele Cruciani, Loriano Storchi

**Affiliations:** ^1^ Molecular Horizon srl via Montelino 30 Bettona (Perugia) 06084 Italy; ^2^ Department of Chemistry, Biology and Biotechnology University of Perugia Perugia Italy; ^3^ Translational Informatics Division, Department of Internal Medicine University of New Mexico School of Medicine Albuquerque New Mexico USA; ^4^ Dipartimento di Farmacia Università G. D'Annunzio Chieti Italy; ^5^ Molecular Discovery Ltd Hertfordshire UK

**Keywords:** drug discovery, machine learning, medicinal chemistry applications, molecular descriptors, molecular interaction fields

## Abstract

Molecular interaction fields (MIFs), describing molecules in terms of their ability to interact with any chemical entity, are one of the most established and versatile concepts in drug discovery. Improvement of this molecular description is highly desirable for in silico drug discovery and medicinal chemistry applications. In this work, we revised a well‐established molecular mechanics' force field and applied a hybrid quantum mechanics and machine learning approach to parametrize the hydrogen‐bonding (HB) potentials of small molecules, improving this aspect of the molecular description. Approximately 66,000 molecules were chosen from available drug databases and subjected to density functional theory calculations (DFT). For each atom, the molecular electrostatic potential (EP) was extracted and used to derive new HB energy contributions; this was subsequently combined with a fingerprint‐based description of the structural environment via partial least squares modeling, enabling the new potentials to be used for molecules outside of the training set. We demonstrate that parameter prediction for molecules outside of the training set correlates with their DFT‐derived EP, and that there is correlation of the new potentials with hydrogen‐bond acidity and basicity scales. We show the newly derived MIFs vary in strength for various ring substitution in accordance with chemical intuition. Finally, we report that this derived parameter, when extended to non‐HB atoms, can also be used to estimate sites of reaction.

## Graphical abstract

Approximately 66,000 molecules were subjected to density functional theory calculations. The extracted molecular electrostatic potential has been used to derive new hydrogen‐bonding (HB) energy contributions; this was subsequently combined with a fingerprint‐based description of the structural environment via partial least squares modeling. The newly derived molecular interaction fields vary in strength for various ring substitution in accordance with chemical intuition. Finally, we report that this derived parameter, when extended to non‐HB atoms, can also be used to estimate sites of reaction.

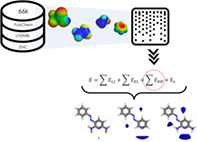

## 1 INTRODUCTION

The use of in silico techniques to predict key molecular properties is nowadays well established in drug discovery.^1^ The reliability of these techniques is mainly based on their ability to estimate molecular properties with realistic chemical sense that can account for experimental properties. Therefore, research on new and more reliable in silico molecular descriptors for typical drug‐scaffolds is still active and different approaches have been proposed in the last decades.^2^, ^3^, ^4^, ^5^, ^6^ In this context, molecular interaction field (MIF)‐based methods aim to describe molecules in terms of how they interact with other chemical entities, rather than in terms of their chemical structure directly.^7^ Still today, MIFs are one of the most established and versatile concepts in drug design. In ligand‐based design they have been widely used to derive quantitative 3D structure–activity relationship (3D‐QSAR) models to predict binding affinity and pharmacokinetics parameters (e.g., membrane permeability and metabolism), and for virtual screening and pharmacophore modeling; in structure‐based design, they have been used to suggest modifications that improve ligand potency, for virtual screening, and to analyze and estimate protein–ligand and protein–protein interactions (PPIs).^8^, ^9^, ^10^, ^11^, ^12^, ^13^, ^14^, ^15^

A MIF quantifies the spatial variation of the interaction energy between a 3D molecule and a chosen probe that represents a specific chemical type (e.g., water, hydrophobic, amide NH donor, carbonyl O acceptor, carboxylic acid). Usually, in drug design software, the information contained in a MIF is rendered as an isovolume (a region of space which encloses values of the MIF below a certain threshold) and has been further condensed into various numerical descriptors (e.g., VolSurf+^16^) for visualization and ease of interpretation.^17^, ^18^, ^19^ Such interactions are estimated by well‐established molecular mechanics (MM) force fields (GRID^8^ or MOE,^18^ among others^20^, ^21^) which use an energetic potential based on the total interaction energy between a target molecule and a probe and its spatial orientation. Each probe represents a specific chemical group so that chemically specific information can be accumulated about the way in which the target might interact favorably with other molecules. In the GRID force‐field this energy function^8^, ^22^, ^23^, ^24^ is the sum of the energies calculated between the probe, placed at a specific grid point, and every appropriate atom of the target and consists of four terms: Lennard‐Jones (*E*_*LJ*_), electrostatic (*E*_*EL*_), hydrogen‐bonding (*E*_*HB*_), and entropic (*E*_*S*_). Thus, the selected probe is moved between various grid points covering the target, and at each point the energy is computed accordingly to the following equation:

(1) $$E=\sum E_{\mathrm{LJ}}+\sum E_{\mathrm{EL}}+\sum E_{\mathrm{HB}}+E_{s}.$$

The *E*_*HB*_ term is the product of three terms, one based on the distance between the target and the probe (*E*_*r*_) given in kcal/mol, and the other two, both ranging in the interval 0–1, *E*_*t*_, and *E*_*p*_. Both *E*_*t*_ and *E*_*p*_ are dimensionless functions of the angles *t* and *p* made by the hydrogen bond (HB) at the target and the probe atoms respectively. Thus, they describe the orientational dependence of the HBs; for example, *E*_*p*_ assumes a value of 1.0 when the probe is oriented to form the strongest HB possible. Hence, the hydrogen‐bond term is computed as follows:

(2) $$E_{\mathrm{HB}}=E_{r}*E_{t}*E_{p}.$$

According to this definition, the *E*_*HB*_ term, and more specifically the distance dependent part *E*_*r*_, assume relative values in case of interaction with a HB acceptor or donor complementary probe and is parametrized by two values: the strongest hydrogen‐bond attraction energy at the optimum position (*Emin*), and half of the straight‐line distance between donor and acceptor atom pairs which corresponds to the strongest hydrogen‐bond attraction energy (*Rmin*).

In some of the most used force fields,^8^, ^18^, ^22^, ^23^, ^24^ atoms are classified into general classes called “atom types” (e.g., see ATs reported in Table S1) depending on their neighboring atoms, such that each AT represents a specific chemical moiety. In the GRID force‐field, as in most of the used force fields, Emin assumes fixed values for each AT. Such parameterization can be defined as “static”: it does not consider the chemical environment of the atom; a pyridine nitrogen (N:= AT) will always have the same hydrogen bonding parameters regardless of any decoration on the pyridine moiety that might influence the strength of the potential HB. The main reasons for this generalization, especially considering that these force fields were introduced almost 30 years ago, are the speed of the atom classification step, enabling a broad coverage of the drug‐like molecules space with a reasonable number of atom types (in GRID there are 74, including 18 different types of nitrogen and 16 different types of oxygen atom). However, especially when dealing with heteroatoms, such an approximation may fail to accurately define the effect of a nearby chemical substitution on the electrostatic component in the hydrogen bonding contribution, which would be seen via the experimental properties of the moiety. One way to include this chemical effect from nearby structural features of a molecule is to map its electrostatic potential (EP). Indeed, as widely reported in literature, the EP noticeably correlates with HB properties and, more generally, with reactive behavior.^25^, ^26^, ^27^, ^28^, ^29^, ^30^, ^31^

Therefore, starting from the hypothesis that the HB energy is dependent on the environment constituted by neighboring atoms, in this work we present a machine learning and semi‐empirical computational procedure developed to improve the HB description that is implemented as a dynamic contribution in the GRID^8^ force field and used to derive new electronic descriptors which are freely available in VolSurf 3 (VS3 can be downloaded at: https://www.molhorizon.it/software/volsurf3/).

The complete procedure and the results obtained are described in the next sections. In addition, the efficacy of the novel parametrization is demonstrated via correlation to experimental acidic/basic and donating/accepting HB properties (Berthelot and Laurence pK_HB_ database,^32^, ^33^, ^34^, ^35^ and Abraham's hydrogen bonding strength scales^36^, ^37^). Finally, the impact of such dynamic parameterization on derived MIFs is also reported, and proof of concept applications on modern medicinal chemistry approaches are presented. Additional details are available in the Supporting Information (SI).

## 2 METHODOLOGY

A database of approximately 66,000 molecules compounds was built using both combinatorial chemistry approaches and publicly available databases (CheEMBL,^38^ PubChem^39^ additional details are available in SI) with the aim of achieving reasonable coverage of drug‐like space. Subsequently, for all molecules atom‐centered EPs were estimated using density functional theory (DFT) calculations and used to parametrize new dynamic Emin values (*dEmin*) via linear equations. Atoms were classified according to their GRID H‐bond acceptor and donor types (Table 1) and their atomic environment described using a tree‐structured fingerprint.^40^ Subsequently, the partial least squares (PLS)^41^ algorithm was used to correlate specific ATs and their atomic environments to their corresponding *dEmin* values. The obtained PLS models were both internally and externally validated, demonstrating their ability to estimate EP values also for the relevant atom types in unknown molecules. It is maybe important to underline that we used the name *dEmin* (i.e., dynamic Emin) to emphasize that as its value is no more a “static” one, but it considers the chemical environment of the atom; a pyridine nitrogen (N:= AT) will have a different hydrogen bonding parameters depending on the decoration of the pyridine moiety that might influence the strength of the potential HB.

**TABLE 1:** Statistical parameters for the obtained models. AT atom type; chemical description of the atom type; H‐bond type H‐bond donor (D) or H‐bond acceptor (a); atoms number of atoms of the training set; LV number of latent variables considered; R^2^ coefficient of determination for the training set; Q^2^ coefficient of determination for predicted compounds; SDEC standard deviation error in calculation; SDEP standard deviation error in external prediction

AT	Description	H‐bond type	Atoms	LV	R^2^	Q^2^	SDEC (kcal/Mol)	SDEP (kcal/Mol)
N:	sp3 (tertiary) nitrogen, accepting one H‐bond	A	6954	9	0.92	0.88	0.56	0.41
N1:	sp3 (secondary) nitrogen, donating one hydrogen and accepting one H‐bond	A	3941	8	0.91	0.84	0.24	0.49
		D	4776	7	0.96	0.92	0.30	0.53
N2:	sp3 (primary)nitrogen, donating up to two hydrogen and accepting one H‐bond	A	3618	8	0.84	0.71	0.26	0.38
		D	4895	7	0.95	0.92	0.30	0.41
ON	oxygen of nitro or nitroso group, accepting up to two H‐bond	A	4907	8	0.82	0.69	0.26	0.38
N:=	sp2 (aromatic) nitrogen, accepting one H‐bond	A	27,140	12	0.91	0.89	0.35	0.47
N::	sp2 nitrogen with two lone pairs and one double bond	A	472	4	0.89	0.59	0.23	0.12
N:#	sp nitrogen	A	15,798	10	0.72	0.66	0.29	0.32
O1	Alcoholic oxygen atom in sp3 hydroxyl group, capable of donating one hydrogen and accepting up to two H‐bonds	A	1367	6	0.86	0.66	0.30	0.55
		D	1392	7	0.87	0.65	0.29	0.50
OC1	Aliphatic and aryl ether oxygen, accepting one H‐bonds	A	12,725	10	0.74	0.66	0.32	0.44
OC2	Aliphatic ether oxygen, accepting two H‐bonds	A	7100	8	0.81	0.73	0.30	0.44
OC=	Aryl ether oxygen, accepting one H‐bond	A	2527	9	0.89	0.75	0.26	0.46
OES	Tetrahedral ester oxygen, not accepting H‐bonds	A	11,501	10	0.82	0.76	0.28	0.39
OFU	Aromatic furan or oxazole oxygen, accepting one H‐bond	A	6114	9	0.88	0.81	0.26	0.47
OH	Phenolic and carboxy oxygen, capable of donating one hydrogen and accepting up to two H‐bonds	A	4892	7	0.78	0.62	0.29	0.50
		D	4892	7	0.78	0.62	0.29	0.50
O=S	Oxygen bonded only to one central S (sulphones, sulfates, unionized sulfate, sulphonamides), accepting two H‐bonds	A	15,886	10	0.84	0.81	0.24	0.37
OS	Oxygen bonded only to one central S (sulphoxides, unionized sulphonate esters, unionized alkyl sulphinates), accepting two H‐bonds	A	947	4	0.90	0.69	0.25	0.41
O=	Oxygen bonded to one atom (e.g., phosphates arsenates silicates) and accepting up to two H‐bonds	A	13,307	7	0.86	0.83	0.33	0.44
O	sp2 carbonylic oxygen, accepting up to two H‐bonds	A	7811	6	0.90	0.86	0.33	0.61

In the present section, we will detail the methodology adopted, the model building, and validation.

### 2.1 EP from QM calculations

The EP ($V\left(r\right)$) is defined as the electrostatic interaction energy between the molecular charge distribution and the positive unit charge (a proton) located at any point r through the electrical charge cloud generated through the molecule's electrons and nuclei^42^

(3) $$V\left(r\right)=\sum_{A}\frac{Z_{A}}{|R_{A}-r|}-\int \frac{\rho \left(r^{\prime }\right)dr^{\prime }}{\left|r^{\prime }-r\right|},$$

where: $\rho \left(r^{\prime }\right)$ is the electronic density function of the molecule at point $r^{\prime }$, *Z*_*A*_ denotes the nuclear charge placed at *R*_*A*_.

The EP minimum typically lies within the Van der Waals molecular surface.^30^, ^43^ It is a real physical property of a molecule, experimentally measurable by diffraction methods^38^ or computationally estimated via QM calculations.^42^, ^44^, ^45^, ^46^ The accuracy of the computational estimations depends on the “quality” of the chosen method, that is, how well we can approximate the $\rho \left(r^{\prime }\right)$, but an efficiency/accuracy trade‐off has to be found. To this aim, different cost‐efficient ab initio and DFT population analyses for calculating the EP or deriving charges by fitting the MEP (ChelpG,^47^ MK^48^ schemes), have been proposed and their performances evaluated.^27^, ^29^, ^30^, ^46^ In this work, because of the overall dataset size (~66,000 molecules, the list is reported as SI), and the necessity of having a versatile basis set able to describe all the atoms, the B3LYP/SVP^49^, ^50^ level of theory was chosen for EP estimations. We found the SVP basis set to be a good compromise between accuracy and computational cost, indeed the use of more extended basis sets does not seem justifiable in terms of the obtainable results.^51^, ^52^ The GAMESS‐US^53^, ^54^ software was used for EP estimation, after first optimizing the geometry of the input molecules using the semiempirical method AM1; tautomeric states were assigned using the MoKa software.^55^

### 2.2 From QM EP to dEmin

For each molecule of the dataset (66,463 in total), QM calculated EPs were extracted for each atom at each nucleus position (the contribution of each nucleus at its own position is neglected to avoid singularity GAMESS‐US^53^, ^54^). These EP values are converted to *dEmin* values using linear Equations (4) and (5) for each AT; in general, the proposed linear equations have positive intercept and slope for HB‐accepting ATs (Equation (4)), and negative intercept and slope for HB‐donating ATs (Equation (5)).

(4) $$d{\text{Emin}}_{\mathrm{BH}}=m_{\mathrm{BH}}*\mathrm{EP}+q_{\mathrm{BH}}.$$

(5) $$d{\text{Emin}}_{\mathrm{AH}}=-m_{\mathrm{AH}}*\mathrm{EP}-q_{\mathrm{AH}}.$$

In these equations, *m* and *q* are the slope and intercept which are adjusted for each AT to make the different scales comparable, while EP is the calculated EP for a given atom. All the linear equations (reported in Tables S4 of SI) have been derived so that for each AT all the resulting *dEmin* values always fall within an acceptable range according to the GRID Force‐Field (GRID‐FF) parametrization. Thus, each Linear equation is built to compute the new *dEmin* parameter for the GRID‐FF and this parameter used as the dependent variable Y for training the PLS^41^ regression models. The independent X variables come from a tree‐structured molecular fingerprint. Specifically, for each atom, the molecular environment is described by a tree‐structured molecular fingerprint with a length of 10 bond distances in an analogous fashion to that successfully used by Xing and coworkers for modeling pK_a_.^40^ Using this approach, 22 PLS models were built relating atomic environment to *dEmin* for the HB GRID atom types for which enough data was available; other models we also built to predict *dEmin* for other GRID atom types (see Section 4.3). Each PLS model is then used to compute the *Emin* parameter dynamically (*dEmin*) to be used by the GRID‐FF to calculate the hydrogen‐bond term of the interaction energy (see Equations (1) and (2)). The machine‐learning approach is therefore used to modulate the hydrogen‐bond term of the GRID force field depending on the chemical environment of the molecule's relevant hydrogen bonding atom type.

The goodness‐of‐fit of the obtained models was evaluated by calculating the coefficient of determination (R^2^) and the predictive power was evaluated using both cross‐validation and external data set validation. The cross‐validated Q^2^ was obtained by random groups cross‐validation (five groups, 20 different partitioning)^56^ and the standard deviation of calculation errors (SDEC) was also evaluated.^41^, ^57^ External validation consisted of projecting a test set of ligands of the whole Protein Data Bank^58^ (2909 candidates). For the test set the EPs were estimated by QM and the *dEmin* values assigned as for the training set. Finally, the standard deviation of prediction errors (SDEP)^41^, ^57^ was evaluated.

## 3 MODELS BUILDING AND VALIDATION

In Table 1, we summarize the key statistical properties of the 22 PLS models obtained. The ability to reproduce the QM derived *dEmin* ranges from R^2^ = 0.72 to R^2^ = 0.96 (Table 1), with an average R^2^ of 0.86 and an average SDEC of 0.30 kcal/mol, indicating that a large amount of variance is predictable by the tree‐structured fingerprints. The number of latent variables (LVs) for each model has been selected trying to maximize the Q^2^. Quite naturally the number of LVs varies as a function of the dimension of the model, going from 4 in the case of relatively small datasets (e.g., N:: and OS) up to 12 in the case of the N:= dataset that consists of more than 27,000 elements (i.e., atoms).

The predictive ability of the models was initially evaluated by internal cross‐validation, resulting in a promising average Q^2^ of 0.76. However, it is well known that supervised multivariate analyses such as PLS may suffer from overfitting, thus external validation is always recommended.^59^ Therefore, the models' ability to predict the *dEmin* of new compounds was evaluated by projecting 2909 external drug‐like candidates. For each compound, atoms were assigned their GRID atom types, the QM estimated EP was calculated, and then the Tree‐structured fingerprints were calculated as described for the training set (see Methodology, subsection 2.2) and used to project the atoms onto the relevant PLS model. The quality of the obtained predictions is summarized in the standard deviation error prediction (SDEP) column in Table 1 for each AT‐model. It can be noted that the obtained values range from 0.12 to 0.61 kcal/mol, demonstrating that the models are also predictive when applied to new compounds.

**FIGURE 1:**
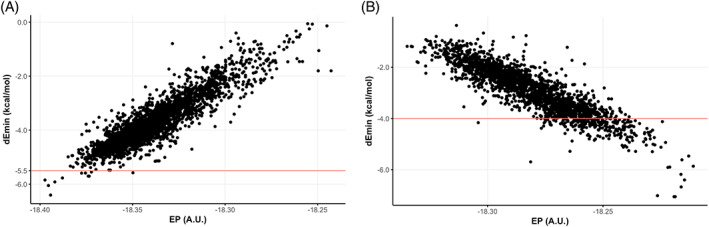
dEmin versus QM electronic potential correlations for (A) the N:= atom type (2711 atoms, R – Pearson = 0.90) and (B) the N1 atom type (2159 atoms, R – Pearson = −0.89) of the test set. The red lines represent values of the traditional, static Emin of the GRID force field, namely −5.5 for N:= and − 4.0 for N1 atom types. dEmin, dynamic Emin [Color figure can be viewed at wileyonlinelibrary.com]

Correlations obtained between the new predicted *dEmin* and the QM EP for the test set are explicitly reported in Figure 1 for the most populated HB‐acceptor and HB‐donor ATs, namely the N:= (sp2 aromatic nitrogen, with 2131 atoms, R^2^ = 0.76, Figure 1A) and the N1 (sp3 secondary amide nitrogen, with 2159 atoms, R^2^ = 0.79, Figure 1B), respectively. Besides the goodness‐of‐fit (see also SI Table S1), it should be emphasized that such correlations would not even have been possible considering the traditional version of the static HB potentials, where for every AT a single, fixed value is assigned (red lines in Figure 1) so no differentiation within atoms of a given AT class was possible.

### 3.1 dEmin correlation to H‐bond basicity and acidity properties

In the pharmaceutical context, HB has a direct influence on the solubility of drugs and on their interaction with their targets.^27^, ^34^, ^35^ In this context, predictive models for HB ability is of high interest for the drug discovery and medicinal chemistry community. Therefore, we decided to test the correlation of the proposed *dEmin* to those experimental hydrogen‐bonding (HB) properties.

**FIGURE 2:**
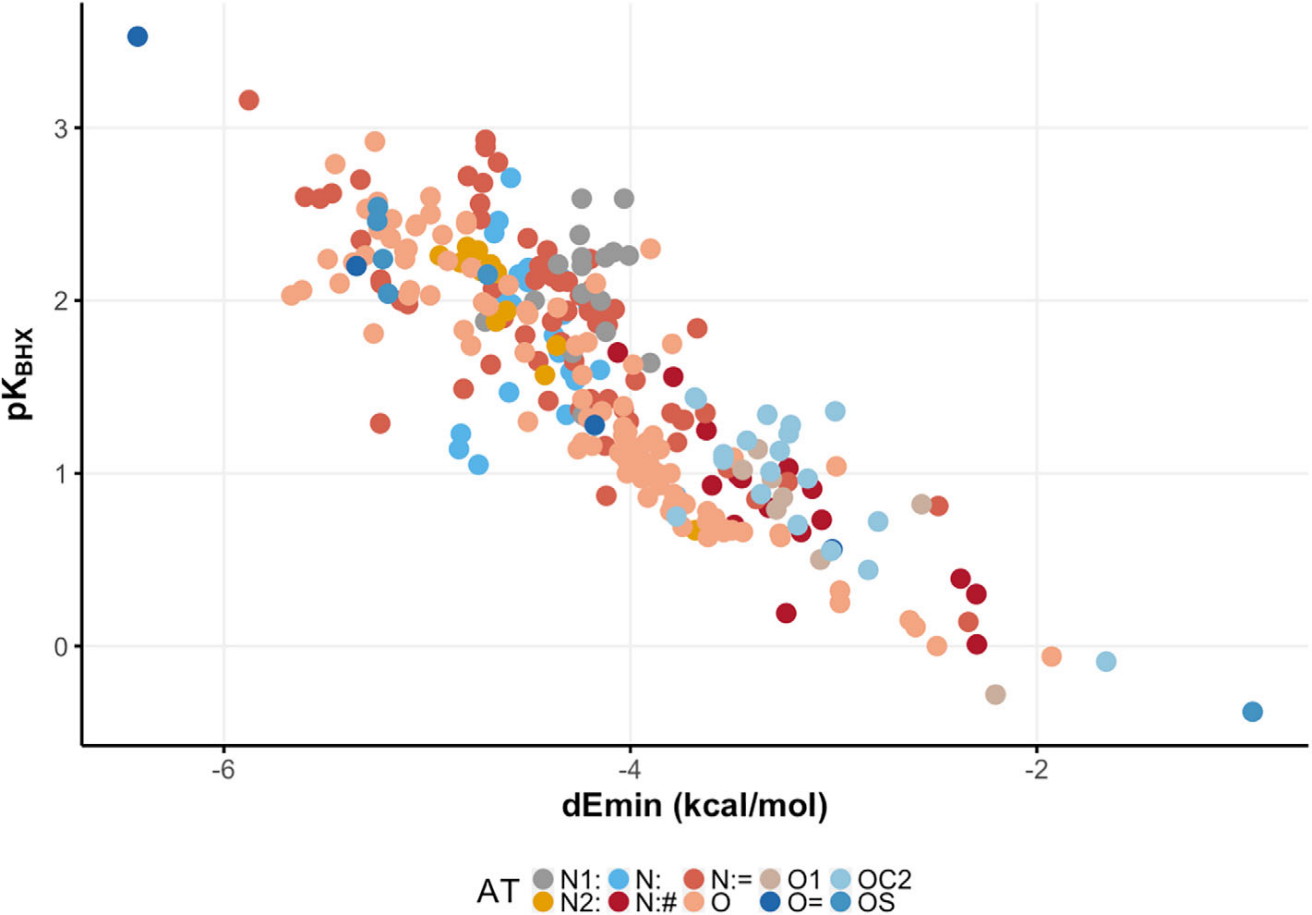
dEmin versus H‐bond basicity scale for the Kenny^27^ dataset (279 atoms, R – Pearson = −0.85). Color palette at the bottom of the picture. dEmin, dynamic Emin [Color figure can be viewed at wileyonlinelibrary.com]

Laurence and colleagues collected several experimental values of HB basicity (pK_BHX_ ^34^, ^35^) to address the still debated issue of describing HB in the context of medicinal chemistry. A collection of these pK_BHX_ values has been reported.^27^ These data were curated as a database, and to avoid concomitant effects only molecules with a single HB acceptor/donor site were retained (the dataset used can be found in SI Table S1). Molecules were projected on our PLS models to obtain the *dEmin* values for each atom of each molecule. As it can be noted in Figure 2, where the experimental pK_BHX_ versus the *dEmin* values have been reported, a good correlation is obtained (279 atoms, Pearson correlation coefficient = −0.85).

Similarly to what has already been shown by Kenny and colleagues^27^ about using the molecular EP as a predictor of pK_BHX_, here we provide a straightforward tool to estimate the pK_BHX_ with good accuracy.

As a proof of concept, we also applied the procedure to acidity scales. In the late 1980s, Abraham and colleagues^36^, ^37^ collected a number of experimental HB basicity ($\log K_{B}^{H}$) and acidity ($\log K_{A}^{H}$) values of common organic molecules, with the aim of obtaining scales of solute HB ability, that are still widely used today for drug design purposes. We took Abraham's databases, curated them as previously described to avoid concomitant effects, and molecules of the database were projected on the relevant AT PLS models to obtain *dEmin* values. The final database used is reported in Supporting Information, Tables S2 and S3. As shown in Figure S2, *dEmin* values successfully correlates with experimental HB basicity (Figure S2 A, 140 atoms) and acidity (Figure S2 B, 89 atoms) properties, with Pearson correlation coefficient equal to −0.90 and −0.86, respectively.

A figure of merit is that, while EP intrinsically refers to a specific AT so that a direct comparison among different ATs is not possible, *dEmin* values refer to a unique scale. This allows a straightforward comparison of *dEmin* values among different ATs and to explore correlations to physical–chemical properties of interest, such as $K_{B}^{H}$ and $K_{A}^{H}$. Once again, it is important to underline that such correlation would not have been possible considering the static version of the HB potential used in traditional force fields.

## 4 DRUG DESIGN AND MEDICINAL CHEMISTRY APPLICATIONS

In the following paragraph, we report three real‐world examples related to drug design and medical chemistry applications. To demonstrate the predictive power of the models, in the following examples we are only considering molecules not included in the training set.

### 4.1 Case study I: Chemically aware MIFs for functionalization of a drug candidate for COVID‐19

As already mentioned, the spatial variation of the interaction energy derived using the new *dEmin* can be quantified using MIFs. One of the most popular ways to represent the information contained in a MIF is the rendering of isovolumes. As a result, MIFs are represented as 3D objects.^18^, ^60^, ^61^ For this reason, any change in the interaction energy value will be reflected in the obtained MIFs. In order to evaluate if the proposed parametrization is in accordance with the expected chemical behavior, as well as the impact on possible medicinal chemistry and drug design applications, in this example we used the *dEmin* values to compute (see Equation (2)) and represent the MIF.

**FIGURE 3:**
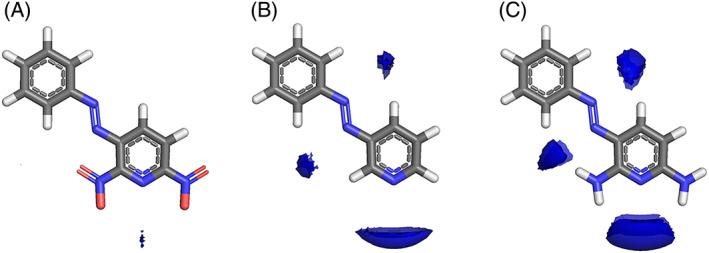
MIFs for phenazopyridine derivatives (A—Deaminated and nitro substituted phenazopyridine B—Deaminated phenazopyridine C—phenazopyridine). The energy values of the isocontour surfaces chosen for H‐bond donating probe (“N1,” blue fields) was −4.0 kcal/Mol [Color figure can be viewed at wileyonlinelibrary.com]

Phenazopyridine is a urinary tract analgesic used for the short‐term management of urinary tract infections, surgery, or injury to the urinary tract, but in a recent study, it was reported among the approved drugs with putative activity against SARS‐CoV‐2 targets.^62^ Imagining a chemist who wants to further optimize the DMPK (Drug Metabolism and Pharmacokinetics) properties of phenazopyridine by small moiety substitution (i.e., drug design approach), we computed MIFs for phenazopyridine and two derivatives with substituents accounting for different electronic effects (Figure 3). Chemical sense would suggest that a phenazopyridine substituted with an electron‐withdrawing group on the pyridine ring would withdraw electron density from the pyridine N atom, therefore reducing its ability to accept a HB from a HB donor. Therefore, one would expect a HB donor MIF (e.g., From the N1 amide NH probe) to be weaker (smaller volume at the equivalent isocontour level) when interacting with a nitro‐substituted phenazopyridine (Figure 3A) versus phenazopyridine. Without any substituents on the ring results in a stronger interaction (Figure 3B), and phenazopyridine shows a yet stronger interaction the electron‐donating substituents (Figure 3C). While the MIFs are describing the overall interaction according to Equation (1), at the isocontour level of −4 kcal/mol the interaction energy for the N1 probe will be dominated by the hydrogen bonding term which itself is derived from the new *dEmin* value.

### 4.2 Case study II: Novel VS3 molecular descriptors for the prediction of an ADMET property

VolSurf+^16^, ^63^ is a computational procedure designed for a fast generation of quantitative molecular descriptors. In the past, VolSurf+ has been applied with success in several drug development projects.^64^, ^65^, ^66^ We used the new descriptors to develop a machine‐learning model for predicting the fraction of a drug dose that is excreted unchanged in urine. This property belongs to the ensemble of mechanisms and biological processes that describe the adsorption, distribution, metabolism, excretion, toxicity (ADMET) of a drug^67^ and its assessment is required by regulatory agencies such as the FDA.^68^ It represents the degree of renal clearance with respect to the overall human body clearance of the drug, which includes other routes (e.g., metabolic, biliary, etc^69^). Depending on whether a drug is prevalently found in urine with its chemical structure unmodified or not is important for a number of reasons. For example, a drug that is prevalently found unchanged in urine undergoes neither phase I nor phase II metabolism in the gut and liver (or at least a negligible amount). The bile (and the enterohepatic recirculation) is not the primary route of excretion of the drug either. The drug therefore has a lower risk of incurring drug–drug interactions because it is not a substrate of the metabolism enzymes. Consequently, it is of paramount importance to know this property for drug candidates in the early development stage because it can be used to drive the selection of optimal compounds and to shrink the number of experiments that need to be performed.

**FIGURE 4:**
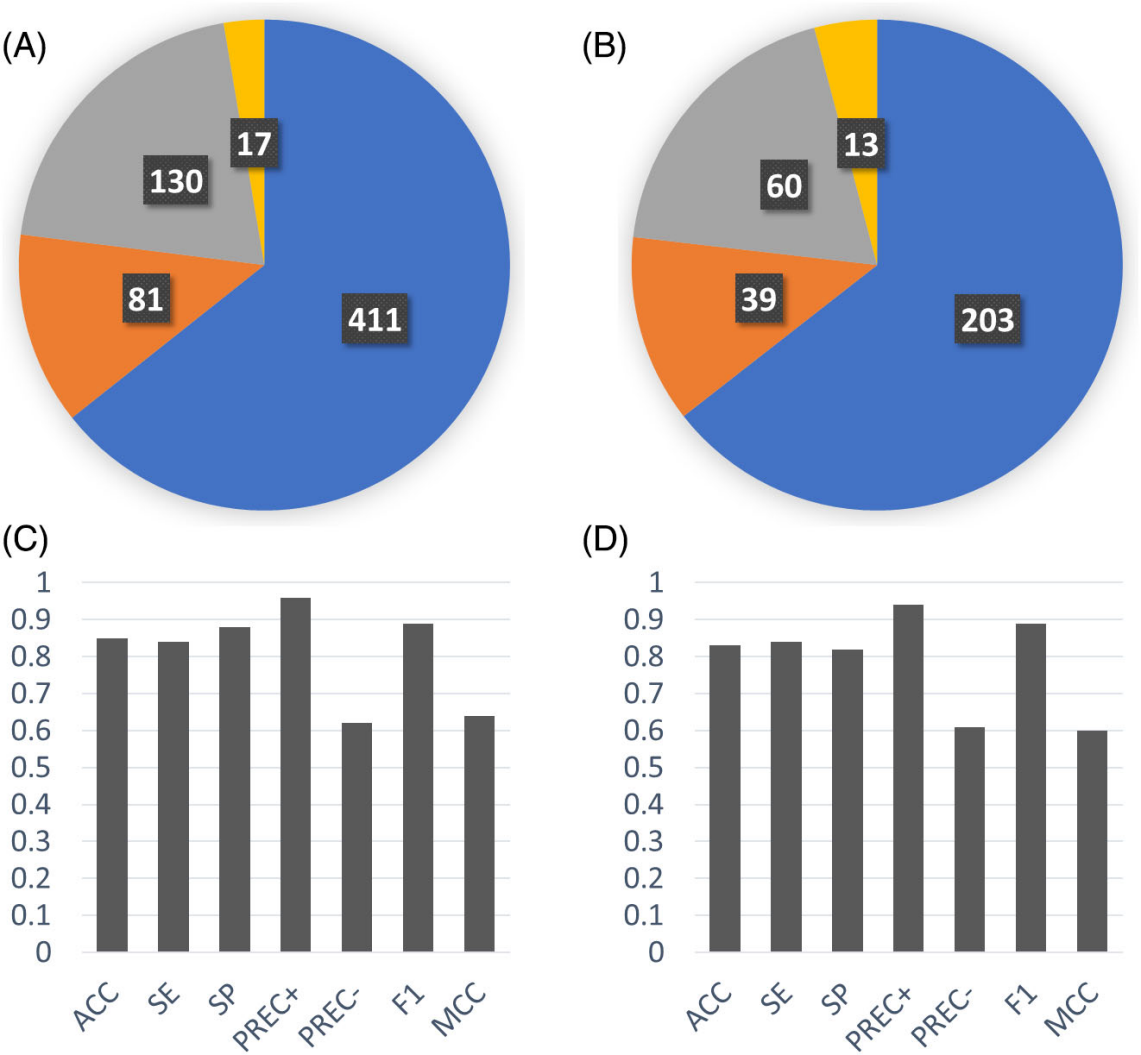
Summary of the “fraction of drug dose excreted unchanged in urine” model performance. (A) Pie chart depicting the training set confusion matrix. (B) Pie chart depicting the test set confusion matrix. (C) Metrics showing the model performance in fitting (prediction of training set molecules). (D) Metrics showing the model performance in validation (prediction of test set molecules). In the confusion matrix pies, colors indicate the different predictions: True positives in blue, false negatives in orange, true negatives in gray and false positives in yellow. In the bar plots, the metrics described are the following: ACC, accuracy; F1, f1‐score; MCC, Matthew's correlation coefficient; PREC+, positive precision; PREC‐, negative precision; SE, sensitivity; SP, specificity [Color figure can be viewed at wileyonlinelibrary.com]

VolSurf^3^(Molecular Horizon srl, Perugia Italy), the VolSurf+ version including the proposed dEmin parametrization, embeds types of descriptors that are well suited for describing/predicting ADMET properties. Here we show the development of a computational model that can be used in early stages of drug discovery to forecast the fraction excreted unchanged in urine simply starting from chemical structures. The experimental data for 954 drugs was collected from a publication by Benet et al.,^70^ converted into categorical values and used for training and validating a machine learning model based on the random forest algorithm^71^ using the scikit‐learn software package^72^ (see SI for details). The model performance in reproducing the training data (fitting) and in predicting the test data (external validation) are depicted in Figure 4. The confusion matrix of training and test sets are shown in Figure 4A,B respectively, whereas the prediction metrics for training and test sets are reported in Figure 4C,D, respectively. As it can be seen, the model developed with VolSurf^3^ descriptors accurately predicts the fraction excreted unchanged in urine and confirms their applicability to the study of other ADMET properties.

### 4.3 Case study III: Novel VS3 electronic descriptors for site of reaction estimation

In the previous sections, we described the derivation of the new *dEmin* parameters from the QM calculated EP centered on the nucleus of atoms involved in HB, and showed how these values coupled with descriptors describing the atomic environment could be used to derive PLS models to predict such values more generally. The values were then used as parameters for the HB term of the GRID force field to predict molecular interaction energies. We realized that the same approach could be used to derive descriptors for all of the GRID atom types (see Table S5 for details), and might give an indication of which atoms are more reactive. Since these descriptors are no longer describing the *Emin* parameter in the GRID force field, we are renaming them as GRID charges (GC) and they are reported as such in the VolSurf^3^ software.

Late‐stage functionalization (LSF) is an emerging synthetic strategy in the drug discovery scenario.^73^ Through C–H functionalization of drug leads or intermediates, new analog are readily generated in a few synthetic steps, with clear benefits over de novo syntheses. The main pitfall of LSF is that multiple regioisomeric products are generated and therefore the reaction follow‐up consists of time‐consuming and laborious purification and structure confirmation steps. In this context, chemically aware data processing can expedite the process of interpreting analytical methods developed for the batch analysis of high‐throughput reaction screenings. An excellent example was recently reported by Yao and collaborators^74^ who coupled LC‐HR‐MS/MS with automated, chemically aware data processing (Mass‐ChemSite, Molecular Discovery Ltd, London UK) to rapidly provide information about reaction conversion, numbers of product isomers, and the more probable sites of reactivity. The GC descriptor outlined above also describes the electronic properties of carbon atoms, and in this case study we use the results presented by Yao and co‐workers to demonstrate its utility to predict the products of different LSF approaches for a set of marketed drugs.

Risperdal and methotrexate underwent acid‐promoted electrophilic halogenation, a widely used strategy for introducing halogen functionalities. The VolSurf^3^ GC descriptor can be used to identify the most electron‐rich carbon atom, which is the optimal candidate for electrophilic halogenation. As reported in Table 2, there is a perfect agreement between the most electron‐rich carbon atom as predicted by the GC descriptor and the experimental site of reaction, potentially providing a more accurate estimation of the reaction site with respect to that proposed by LC–MS and the data‐driven approach recently proposed by Yao and co‐workers.

**TABLE 2:** Predicted and experimental sites of reaction prediction as in Reference 68 compared with VolSurf^3^ electronic description (GRID charges, GC). Highlighted in bold, the molecular moiety of possible sites of reaction proposed in Reference 68. Electron‐poor molecular moieties are highlighted in red, electron‐rich in blue

Substrate	Reaction	Predicted	Experimental
Risperdal	Electrophilic halogenatation	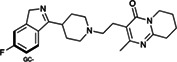	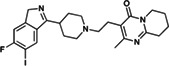
Methotrexate	Electrophilic halogenatation	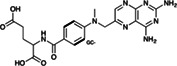	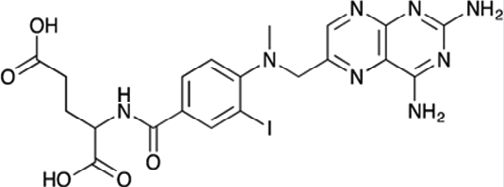
Voriconazole	Acid‐promoted electrophilic bromination	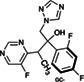	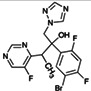
Pioglitazone	Baran‐Minisci reaction with different alkylsulfinate Diversinate	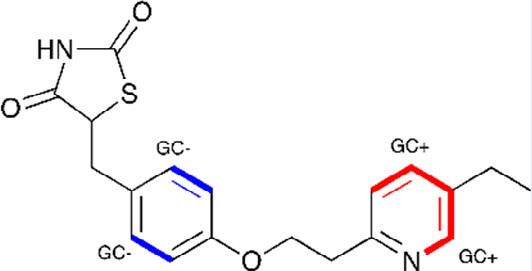	

Another widely employed reaction for LSF is the addition of alkyl radicals to heteroaromatic rings, often referred to as the Minisci reaction.^75^ Voriconazole underwent a recently proposed variant of the Minisci reaction, namely an acid‐promoted electrophilic bromination. Also, in this case the GC descriptor identified as the most electron‐rich carbon site the one found experimentally as the site of reaction.

Clearly, the final product always depends on the nature of all the reagents involved, and the site of reaction is not an intrinsic property. For instance, pioglitazone was subjected to LSF via a Baran‐Minisci^75^, ^76^ reaction with different alkylsulfinate Diversinate salts and reaction conditions. As expected, the final product depended on the electronic nature of the radicals: electron‐deficient ones (e.g., trifluoromethyl and 1‐[trifluoromethyl]cyclopropyl) attacked the electron‐rich para‐disubstituted benzene ring, while the electron‐rich ones (isopropyl) attacked the electron‐deficient pyridine ring.^74^ In this scenario, the GC descriptor can be used to identify the electron‐deficient ring and the electron‐rich ring (highlighted in Table 2 in blue and red, respectively), thus enabling the prediction of the site of reaction.

As also underlined by Yao and co‐workers,^74^ the main advantages of integrating such automated but chemically aware analysis in the interpretation of analytical methods is that they can be used for batch processing of high‐throughput chemistry screens. The main innovation, with respect to the workflow proposed by Yao, is that by using the GC descriptor, the exact site of reaction can be readily identified. We therefore speculate that it can be used in Mass‐ChemSite (Molecular Discovery Ltd, London UK) and analog approaches to further refine and prioritize the estimated site of reactions, in the same way as which the MetaSite prediction algorithm can be used to refine and prioritize the site of metabolism assigned by Mass‐MetaSite, which may be ambiguous within a particular mass fragment.

Finally, two other marketed drugs were investigated by Yao and co‐workers, sumatripan and indomethacin. In this case C–H borylation employing an Ir‐based catalyst was chosen as the LSF reaction. Using such an inorganic catalyst, the site of reaction is determined more by steric effects than electronic ones; hence, in this case it is not appropriate to use electronic descriptors to estimate the possible reaction product.

## 5 CONCLUSIONS

Through a hybrid quantum mechanics and machine learning approach, we have proposed a novel parametrization of the hydrogen‐bond potentials that can be implemented in traditional force fields to obtain more reliable and chemically aware MIFs. An extension of the approach led to in silico descriptors that can be used to estimate the site of reaction.

We used DFT calculations on a diverse set of 66,000 molecules to extract the EP at each atom and derived new dynamic hydrogen‐bond potential values (*dEmin*) through atom type specific equations. Then, tree‐structured fingerprints were used to describe the atomic environment and PLS were used to establish a relationship between this atom environment description and the *dEmin* parameter. Unlike EP which has an atom‐specific scale (i.e., AT specific scale), *dEmin* can simultaneously describe different HB donor/acceptor atom types coherently with their experimental behavior, a key requirement for usability in drug design and medicinal chemistry applications. A comparison with experimental acidity and basicity scales for organic compounds showed an inverse correlation of −0.85 to −0.9, demonstrating that *dEmin* is describing well the HB acidity and basicity, in line with other authors (e.g., Kenny and co‐workers^77^) who have also shown that EP correlates with H‐bonding properties referring to the EP close to the vdW surface.

Our primary goal was to optimize the GRID force field parametrization, and we believe we have demonstrated that more chemically aware MIFs can be generated from the proposed *dEmin*; using the static version of the *Emin* all of the results presented here would not have been possible (e.g., see Figure 3 or Figure 1). Moreover, a novel descriptor to estimate atomic reactivity via electronic properties has been introduced, and it is application demonstrated using proof‐of‐concept examples in the field of medicinal chemistry. This descriptor has been implemented in the newest version of VolSurf (VS3), which is freely available for non‐profit research institutions.

Overall, we believe that such novel in silico parameterization will enhance the quality of the drug design studies based on the traditional force fields and derived MIFs and molecular descriptors, ultimately providing medicinal chemists with a more accurate description of the compounds that they strive to optimize.

## CONFLICT OF INTEREST

The authors declare no competing financial interest.

## Supporting information


**Appendix** S1: Supplementary Information

## Data Availability

Data sharing is not applicable to this article as no new data were created or analyzed in this study.
