# Supplementary material for: Combining machine learning and quantum mechanics yields more chemically aware molecular descriptors for medicinal chemistry applications
Source: J Comput Chem. 2021 Aug 19;42(29):2068–78. doi: 10.1002/jcc.26737 (PMC9291213; doi:10.1002/jcc.26737)
Supplement: Supplementary file 1 — Appendix S1: Supplementary Information [file JCC-42-2068-s001.pdf]

SUPPORTING INFORMATION

Combining Machine Learning and Quantum Mechanics Yields More Chemically-Aware Molecular Descriptors for Medicinal Chemistry Applications

Sara Tortorella[1*], Emanuele Carosati[2], Giulia Sorbi[1], Giovanni Bocci[3], Simon Cross[5], Gabriele Cruciani[2], Loriano Storchi[4, 5*]

[1] Molecular Horizon srl, Via Montelino 30, 06084 Bettona - Perugia, Italy

[2] Department of Chemistry, Biology and Biotechnology, University of Perugia, Via Elce di Sotto 8, 06123 - Perugia, Italy

[3] Translational Informatics Division, Department of Internal Medicine, University of New Mexico School of Medicine, Albuquerque, New Mexico 87131, NM, USA

[4] Dipartimento di Farmacia, Università G. D'Annunzio, Via dei Vestini 31, 66100 Chieti, Italy

[5] Molecular Discovery Ltd, Centennial Park, WD6 3FG Borehamwood, Hertfordshire, United Kingdom

*e-mail: loriano@storchi.org, sara@molhorizon.it

DATASET USED

To build the model described in the paper we used dataset size of ~66,000 molecules, more than 90% of the dataset is made of publicly available structures https://doi.org/10.5281/zenodo.4555770

A

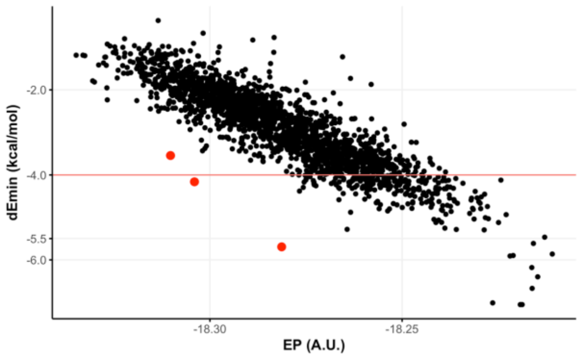

B

CDA_11 **CDD_11** **ROI_4**

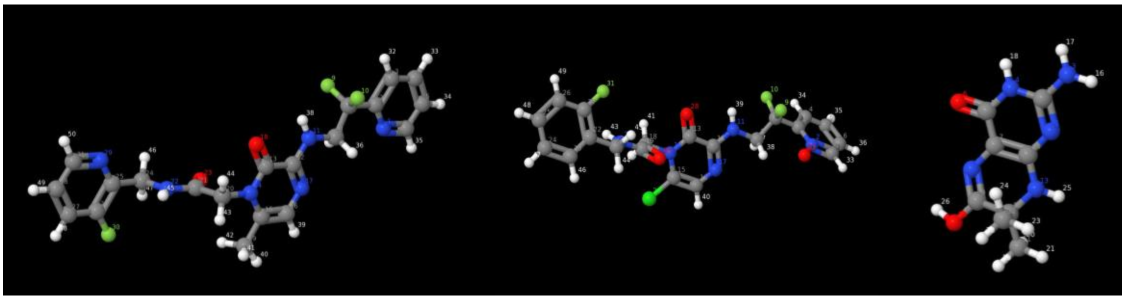

Figure S1 (A) dEmin vs QM EP correlation for the N1 atom type of the test set. In red, atoms belonging to ligands CDA, CDD, ROI. (B) Structures of ligands CDA, CDD, ROI.

MODELS BUILDING: CONSIDERATIONS ON POSSIBLE OUTLIERS

Outliers from the observed correlation can be rationalized with two considerations. First of all, the fingerprint description is undoubtedly more detailed than the only AT classification, thus leading to significant improvements in terms of wider representation of chemical spaces, better fit to the training set, and more accurate prediction on the test set (Table 2 and Figure 1).[1] However, the MEP can be seen as an electronic picture of the whole molecule, while the fingerprint, having a limited length, might not describe necessarily all of it and this could account for discrepancies especially for bigger molecules. Secondly, the work principles of the proposed PLS projection are: (i) a recognition of the scaffold (as described by the fingerprint) and (ii) association to an estimation of the dEmin, accordingly to what the model has learnt from the training set. That is, if the projected scaffold was not covered in the training set, the estimation is likely to be wrong. For instance, in the N1 correlation (Figures 1B and S1), among the most underestimated we find CDA and CDD, both of them containing a fluorine atom in γ position of the aminic atom that may influence the HB ability in a fashion not covered, thus not correctly estimated, in the training set. For these reasons, the models can be periodically enriched in structures with the aim of covering the full chemical space thus increasing the predictive efficacy.

Table S1 Data used for demonstrating the correlation between experimental hydrogen-bond basicity values and the proposed dEmin. AT atom type; pK_{BHX} experimental equilibrium constant for acid-base complexation (as in Kenny's database, 2016[2]). https://zenodo.org/record/4091341#.X4hmB5MzZTY

Table S2 Data used for demonstrating the correlation between experimental hydrogen-bond basicity values and the proposed dEmin. AT atom type; K_{BH} experimental equilibrium constant for acid-base complexation (as in Abraham's database, 1990[3]). https://zenodo.org/record/4091341#.X4hmB5MzZTY

Table S3 Data used for demonstrating the correlation between experimental hydrogen-bond acidity values and the proposed dEmin. AT atom type; K_{AH} experimental equilibrium constant for acid-base complexation (as in Abraham's database, 1989[4]). https://zenodo.org/record/4091341#.X4hmB5MzZTY

A B

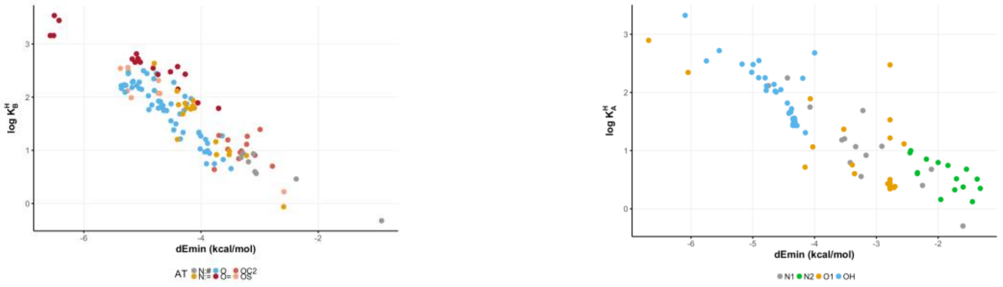

Figure S2 Novel dEmin versus H-bond basicity scale (A: 140 atoms; R – PEARSON = -0.90 ; Colour palette: N:# grey, N:= yellow, O cyan, O= red, OC2 salmon, OS pink) and H-bond acidity scale (B: 8 atoms; R – PEARSON = -0.86; Colour palette: N1 grey, N2 green, O1 yellow, OH cyan). Source: Abraham 1989, 1990.[3,4]

AT	HB type	Slope m	Intercept q	dEmin range of values
N:	HB-acceptor	45.45	828.80	Min = -6.491<br>Max = 0.000
N1:	HB-acceptor	45.45	828.80	Min = -5.310<br>Max = 0.000
N1:	HB-donator	-45.45	-833.80	Min = -9.302<br>Max = -0.690
N2:	HB-acceptor	45.45	829.80	Min = -5.767<br>Max = 0.000
N2:	HB-donator	-45.45	-833.80	Min = -7.476<br>Max = -0.233
ON	HB-acceptor	45.45	1010.80	Min = -4.709<br>Max = 0.000
N:=	HB-acceptor	45.45	828.80	Min = -6.846<br>Max = 0.000
N::	HB-acceptor	45.45	828.80	Min = -3.297<br>Max = 0.000
N:#	HB-acceptor	45.45	831.30	Min = -5.327<br>Max = 0.000
O1	HB-acceptor	45.45	1010.80	Min = -4.718<br>Max = 0.000
O1	HB-donator	-45.45	-1016.80	Min = -7.238<br>Max = -1.282
OC1	HB-acceptor	45.45	1010.80	Min = -3.497<br>Max = 0.000
OC2	HB-acceptor	45.45	1010.80	Min = -4.350<br>Max = 0.000
OC=	HB-acceptor	45.45	1010.80	Min = -2.645<br>Max = 0.000
OES	HB-acceptor	45.45	1010.80	Min = -3.644<br>Max = 0.000
OFU	HB-acceptor	45.45	1009.80	Min= -2.578<br>Max = 0.000
OH	HB-donator	-45.45	-1016.80	Min = -3.069<br>Max = 0.000
OH	HB-acceptor	45.45	1010.80	Min = -7.463<br>Max = 0.000
O=S	HB-acceptor	45.45	1010.80	Min = -5.828<br>Max = 0.000
OS	HB-acceptor	45.45	1011.80	Min = -6.385<br>Max = -0.777
O=	HB-acceptor	45.45	1011.80	Min = -6.779<br>Max = 0.000
O	HB-acceptor	45.45	1010.80	Min = -7.180<br>Max = 0.000

Table S4: Slope and intercept for each AT as defined in equations 4 and 5. For each AT we determined a slope and an intercept so that all the dEmin values will be an acceptable range for the GRID force field.[5] Note the maximum allowable value of dEmin is always forced to be zero.

AT	Slope m	Intercept q
C3	17.18	252.45
C2	17.18	252.45
C2=	17.18	252.45
C1	17.18	252.45
C1=	17.18	252.45
CH	17.18	252.45
C1#	17.18	252.45
C0	17.18	252.45
C=	17.18	252.45
C	17.18	252.45
C#	17.18	252.45
C:#	17.18	252.45
BR	23.78	4180

CL	9.8	630.3
F	21.78	576.4
F3	21.78	576.4

Table S5: Slope and intercept for each AT as defined in equations 4 and 5 for AtomTypes non included in Table S4 and used to define the GC values used in Section 4.3

CASE STUDY II: THE MODEL DEVELOPMENT WORKFLOW

The chemical structures were retrieved from the drug database DrugCentral[6,7] as sdf format and imported into VolSurf by keeping all default settings except for the protonation that was fixed to pH=7.4. A total of 121 molecular, physiochemical and ADME descriptors were computed and exported. Fraction excreted unchanged in urine values were coded into categories by fixing a threshold at 50 %. 220 drugs having values ≥ 50 % were classified as poorly metabolized and 734 drugs having values < 50 % were classified as extensively metabolized. Successively, the categorical property values were matched with the previously generated descriptors for each drug. Data was split into training and test set by random selection and by keeping even percentages across the two categories (see **Figure S2**): 67% of both poorly and extensively metabolized drugs compose the training set; the remaining 33% of both poorly and extensively metabolized drugs compose the test set. The model was trained with the random forest algorithm available in scikit-learn.[8] Algorithm parameters were manually adjusted to achieve optimal performances for both training and test sets (see **Table S4** for details). Finally, the model performances in fitting and validation were evaluated (see **Figure 4**).

Table S6. The parameters used for building the random forest models and their values.

Parameter	Value
bootstrap	TRUE
class_weight	balanced
criterion	entropy
max_depth	4
max_features	log2
max_leaf_nodes	15
min_impurity_decrease	0
min_impurity_split	None
min_samples_leaf	1
min_samples_split	2
min_weight_fraction_leaf	0
n_estimators	60
oob_score	FALSE
random_state	666
warm_start	FALSE

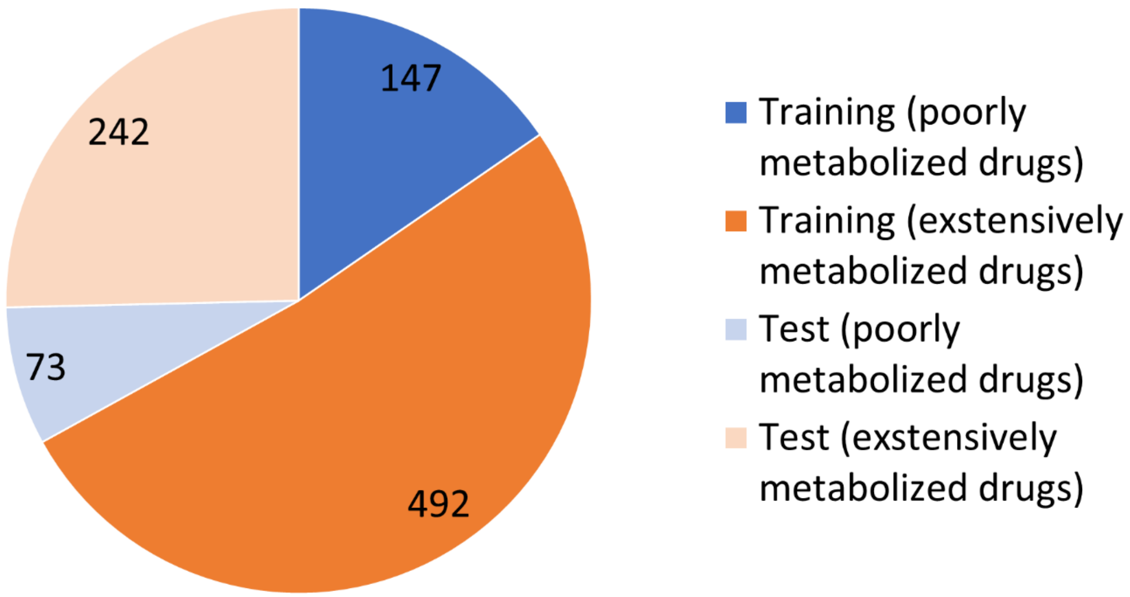

Figure S3. Training and test set composition. For both poorly and extensively metabolized classes, the number of drugs composing each set is 67% for the training and 33% for the test.

REFERENCES

(1) Xing, L.; Glen, R. C.; Clark, R. D. *J. Chem. Inf. Comput. Sci.* **2003**, *43*, 870–879.

(2) Kenny, P. W.; Montanari, C. A.; Prokopczyk, I. M.; Ribeiro, J. F. R.; Sartori, G. R. *J. Med. Chem.* **2016**, *59*, 4278–4288.

(3) Abraham, M. H.; Grellier, P. L.; Prior, D. V; Morris, J. J.; Taylor, P. J. *J. Chem. Soc. Perkin Trans. 2* **1990**, *12*, 521.

(4) Abraham, M. H.; Grellier, P. L.; Prior, D. V; Duce, P. P.; Morris, J. J.; Taylor, P. J. *J. Chem. Soc. Perkin Trans. 2* **1989**, 699.

(5) Wade, R. C.; Clark, K. J.; Goodford, P. J. *J. Med. Chem.* **1993**, *36*, 140–147.

(6) Ursu, O.; Holmes, J.; Knockel, J.; Bologa, C. G.; Yang, J. J.; Mathias, S. L.; Nelson, S. J.; Oprea, T. I. *Nucleic Acids Res.* **2017**, *45*, D932–D939.

(7) Ursu, O.; Holmes, J.; Bologa, C. G.; Yang, J. J.; Mathias, S. L.; Stathias, V.; Nguyen, D.-T.; Schürer, S.; Oprea, T. *Nucleic Acids Res.* **2019**, *47*, D963–D970.

(8) Pedregosa, F.; Varoquaux, G.; Gramfort, A.; Michel, V.; Thirion, B.; Grisel, O.; Blondel, M.; Müller, A.; Nothman, J.; Louppe, G.; Prettenhofer, P.; Weiss, R.; Dubourg, V.; Vanderplas, J.; Passos, A.; Cournapeau, A.; Brucher, M.; Perrot, M.; Duchesnay, E. scikit-learn Machine Learning in Python https://scikit-learn.org/stable/ (accessed Feb 10, 2020).
